# Validation of the Cancer BioChip System as a 3D siRNA Screening Tool for Breast Cancer Targets

**DOI:** 10.1371/journal.pone.0046086

**Published:** 2012-09-26

**Authors:** Joie N. Marhefka, Rula A. Abbud-Antaki

**Affiliations:** Falcon Genomics, Inc., Pittsburgh, Pennsylvania, United States of America; University of South Alabama, United States of America

## Abstract

Genomic studies have revealed that breast cancer consists of a complex biological process with patient-specific genetic variations, revealing the need for individualized cancer diagnostic testing and selection of patient-specific optimal therapies. One of the bottlenecks in translation of genomic breakthroughs to the clinic is the lack of functional genomic assays that have high clinical translatability. Anchorage-independent three-dimensional (3D) growth assays are considered to be the gold-standard for chemosensitivity testing, and leads identified with these assays have high probability of clinical success. The Cancer BioChip System (CBCS) allows for the simultaneous, quantitative, and real time evaluation of multitudes of anchorage-independent breast cancer cell growth inhibitors. We employed a Test Cancer BioChip that contains silencing RNAs (siRNAs) targeting cancer-related genes to identify 3D-specific effectors of breast cancer cell growth. We compared the effect of these siRNAs on colony growth of the hormone receptor positive (MCF7) and Human Epidermal Growth Factor Receptor 2/c- Erythroblastic Leukemia Viral Oncogene Homolog 2 (HER2/c-erb-b2) positive (SK-BR-3) cells on the Test Cancer BioChip. Our results confirmed cell-specific inhibition of MCF7 and SK-BR-3 colony formation by estrogen receptor α (ESR1) and (ERBB2) siRNA, respectively. Both cell lines were also suppressed by Phosphoinositide-3-kinase Catalytic, alpha Polypeptide (PIK3CA) siRNA. Interestingly, we have observed responses to siRNA that are unique to this 3D setting. For example, ß-actin (ACTB) siRNA suppressed colony growth in both cell types while Cathepsin L2 (CTSL2) siRNA caused opposite effects. These results further validate the importance of the CBCS as a tool for the identification of clinically relevant breast cancer targets.

## Introduction

High throughput RNA interference (RNAi) screens have revealed genes essential for the growth of breast cancer cells [1], [2] and sensitivity to current therapies [3], [4], [5]. While these screens identified potential therapeutic targets for overcoming resistance to treatment, their clinical translation has been minimal. Part of the problem is that these assays have been performed using cell lines growing on flat surfaces. Cell lines exhibit extensive chromosomal instability and behave differently depending on the culture conditions. Cellular response to siRNA in these assays is influenced by their attachment to the culture surface and cell-cell contact.

For a long time, anchorage-independent growth assays have been considered to be the gold-standard for chemosensitivity testing for breast cancer [6]. These assays utilize different types of matrices, including soft agar, to inhibit cellular attachment and allow for 3D growth of cells. Transformed tumor cells, but not normal epithelial cells, are capable of growing under these conditions, since they have the innate capability of uncontrollable cell division [7]. Normal epithelial cells depend on cell-cell contact and attachment to a physical support for survival and growth. These unique properties of anchorage-independent growth assays allow for selective chemotoxicity testing of tumor cells in a setting that is 3D, and thus more relevant to the in vivo milieu [6]. Targets identified with these assays have a higher likelihood of clinical success.

It is becoming especially evident that functional genomics screens need to be performed in a 3D anchorage-independent fashion. In a recent study assessing paclitaxel activity in breast cancer cells, 3D tests following a 2D screen revealed differences between the two platforms [3]. Responses to certain inhibitors were observed only in the 3D setting. These findings, combined with the increased clinical relevance of screening cell growth in 3D, reaffirm the benefits of a 3D anchorage-independent platform for identifying novel inhibitors of cancer cell growth.

**Figure 1:**
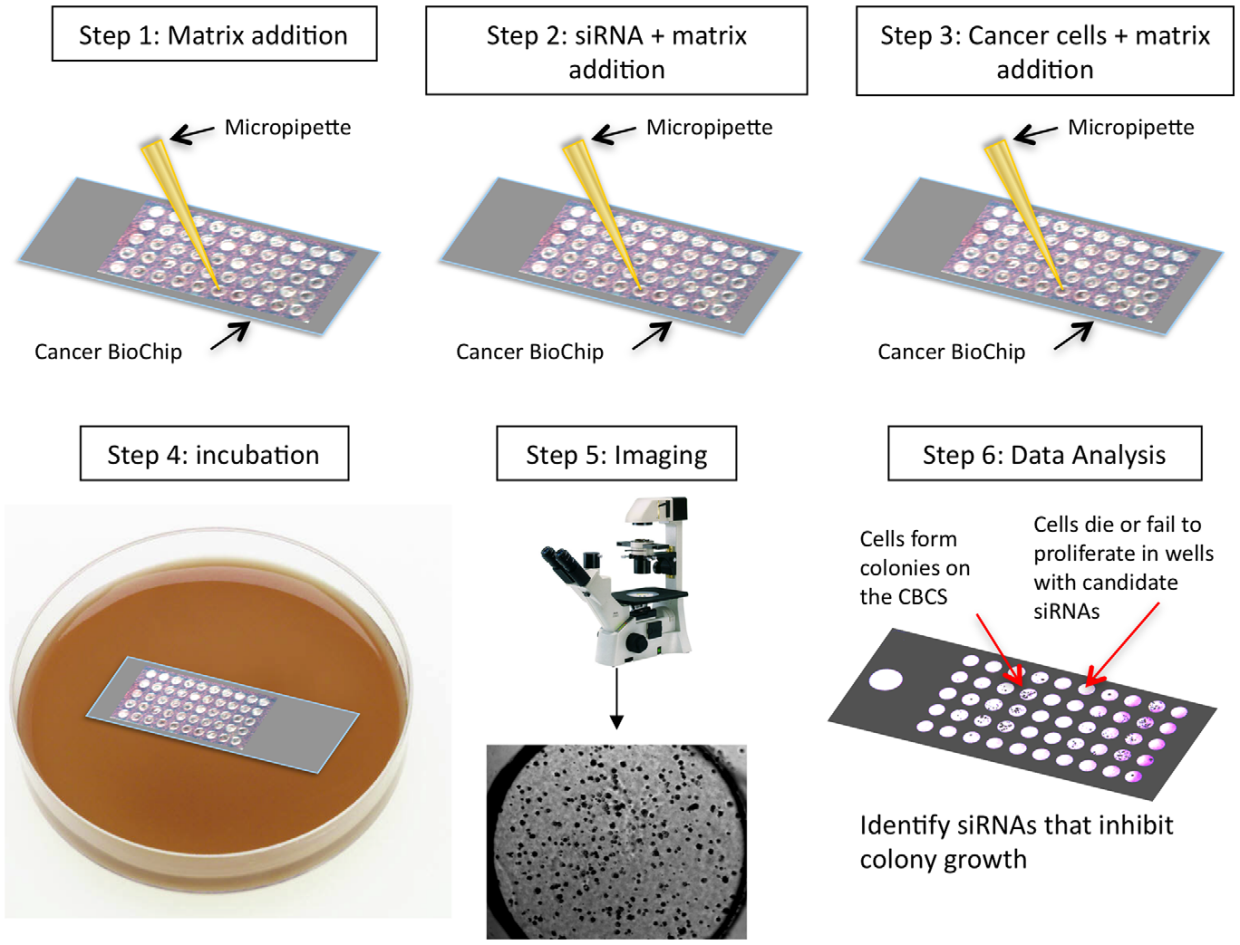
Steps involved in the development of the first-generation Test Cancer BioChip. Shown is the first-generation Cancer BioChip (CBC-1) that is capable of testing the effect of 50 individual siRNAs on colony growth in soft agar. For this study, four CBC-1 were used for screening 40 siRNAs including positive and negative controls. After addition of base agar (step 1), the siRNA was mixed with agar and applied in each well (step 2) followed by cells that were also embedded in agar (step 3). The CBC-1 was fed with medium after a 24-hour incubation (step 4) and growth of colonies was monitored at different time points thereafter using an inverted microscope (step 5). Microscopic image analysis allows for identification of siRNAs that affected colony growth (step 6).

In this paper, we have employed the CBCS (Falcon Genomics, Inc., Pittsburgh, PA; U.S. Patent # 7,537,913 B2 and 8,110,375 B2) as a tool for functional genomics screening of inhibitors of anchorage-independent breast cancer cell growth [8]. The CBCS is a cell-based assay for the high-throughput testing of siRNAs for their ability to inhibit 3D anchorage-independent cell growth. Unique features of the CBCS include using a fast one-step siRNA transfection with live monitoring and quantification of colony growth. When soft agar is used on the CBCS, it selectively tests growth of transformed cells capable of growing in an anchorage-independent fashion. We employed a lower throughput, first generation CBCS (CBC-1) to develop a Test Cancer BioChip (Figure 1) containing siRNA for current druggable breast cancer gene targets [9], and determined whether it can be used for identification and validation of patient-specific targets.

Many of the tested genes are currently either targeted for breast cancer therapy or evaluated in clinical trials. For example, estrogen-related and HER2-related pathways are established targets in breast cancer [10], [11]. The other targets are currently being evaluated for treatment of breast cancer patients in clinical trials including: Ras/Raf/MAPK [12], [13], Phosphoinositide-3-kinase (PI3K) [14], Insulin-like Growth Factor 1 Receptor (IGF1R) [15], c-src (CSK) [16], Heat Shock Protein (HSP90) [17], and the epigenetic modulator Histone Deacetylase (HDAC) [18]. While these and other efficacious therapeutics are currently being developed to target the various pathways associated with breast cancer, methods of predicting patient response to these therapies are essential to advancing the treatment regimen for this disease. We reasoned that patient cells that respond to a particular siRNA on the Cancer BioChip have a high likelihood of responding to drugs that inhibit the targeted gene.

By employing the Test Cancer BioChip, we were able to identify cell-specific and anchorage-independent effects of siRNAs on growth of two breast cancer cell lines: hormone receptor positive MCF7 and HER2 positive SK-BR-3 cells. These include MCF7 inhibition by ESR1 siRNA and SK-BR-3 inhibition by ERBB2 siRNA, as well as suppression of both cell lines by PIK3CA siRNA. Suppression of ACTB also inhibited colony growth in both cell lines, an effect that can only be observed in this 3D anchorage-independent setting. Additionally, we observed a cell type-specific effect of CTSL2 siRNA that is not seen in assays using attached cells. We also observed some minor but statistically significant cell-type specific effects of other siRNAs. These results validate the importance of testing siRNA effects in an anchorage-independent cancer cell growth. Targets identified with this test have a higher likelihood of clinical success.

## Materials and Methods

### Cell Culture

MCF7 and SK-BR-3 cells were obtained from American Type Culture Collection (ATCC, Manassas, VA) in 2008. They were passaged once to generate a tree and cryopreserved until use. Following resuscitation, cells were passaged for less than six months in our laboratory. Authentication of cell lines was performed by ATCC using short tandem repeat profiling.

MCF7 cells were maintained in DMEM (Hyclone Laboratories, South Logan, Utah) supplemented with 10% fetal bovine serum (FBS, Sigma Aldrich, St. Louis, MO) and 1% Antibiotic-Antimycotic (Invitrogen catalog number 15240-062, Carlsbad, California). SK-BR-3 cells were maintained in RPMI (Sigma Aldrich) supplemented with 10% FBS and 1% Antibiotic-Antimycotic. At approximately 70–80% confluency, cells were either passaged using 0.05% Trypsin-EDTA (Hyclone Laboratories) or applied on the Test Cancer BioChip.

### siRNA

Pools of four sequences of siRNA (Accell siRNA, Dharmacon Laboratories, LaFayette, CO) targeting each gene on the Test Cancer BioChip were prepared to give a final concentration of 10 µM on the CBC-1. These include proliferation genes (Ki-67 (MKI67), Aurora Kinase (AURKA), Baculoviral IAP Repeat Containing 5 (BIRC5), Cyclin B1 (CCNB1), v-myb Myeloblastosis Viral Oncogene Homolog (avian)-like 2 (MYBL2)), estrogen related genes (ESR1, Progesterone Receptor (PGR), B-cell CLL/lymphoma 2 (BCL2), Signal Peptide CUB Domain EGF-like 2 (SCUBE2), Estrogen Receptor β (ESR2)), HER2 genes (ERBB2, Growth Factor Receptor-bound Protein 7 (GRB7), invasion genes (Cathepsin L2 (CTSL2), Matrix Metallopeptidase 11 (MMP11), CD68, BCL2-associated Athanogene (BAG1), Glutathione S-transferase mu 1 (GSTM1)), druggable gene targets (IGF1R, Tumor Necrosis Factor Receptor Superfamily, member 10a (TNFRSF10A), Tumor Necrosis Factor Receptor Superfamily, member 10b (TNFRSF10B), Farnesyltransferase CAAX box beta (FNTB), v-raf Murine Sarcoma Viral Oncogene Homolog B1 (BRAF), Mitogen-activated Protein Kinase (MAPK1), PIK3CA, CSK, HSPCA, HDAC1, DNA Methyltransferase 1 (DNMT1), DNA Methyltransferase 1 Associated Protein 1 (DMAP1), and negative controls (ACTB, Glyceraldehyde-3-phosphate Dehydrogenase (GAPDH), Ribosomal Protein Large P0 (RPLP0), Glucuronidase, beta (GUSB), Transferrin Receptor (TFRC), Cyclophilin, Non-Targeting, and no siRNA). Sequences for these siRNAs and accession numbers can be found in the supporting information (Table S1). We also included control siRNA for measurement of transfection efficiency (Accell Green). siRNA concentrations were measured using a Biotek Epoch Spectrophotometer (Winooski, VT).

### Transfection Efficiency

The ability of breast cancer cells to incorporate Accell Green (Dharmacon), a fluorescent siRNA, on the CBC-1 was used to assess transfection efficiency. High transfection efficiency indicates that a large portion of the cells integrated the siRNA, which is essential for an effective siRNA screening assay. Accell Green, at a concentration of 10 µM, was tested, as well as a non-targeting control, which did not exhibit fluorescence. Five replicates were performed for each screen. A z-stack of images was taken of the same region of each well in both bright field and fluorescence using a 10 × microscope objective. Images were acquired using a QICAM (QImaging®, Surrey, BC) mounted on an inverted Motic (Richmond, BC) AE31 microscope using QCapture Pro Imaging Software (QImaging®). Macros written in Image J (National Institutes of Health, Bethesda, MD) were used for analysis. Cells were counted in each image, and those exhibiting fluorescence intensity greater than 2 * SD over the non-targeting control mean were determined to have incorporated the Accell Green. Transfection efficiency for each cell line was expressed as a percentage of cells showing a fluorescent signal. Data is presented as mean transfection efficiency ± SEM.

### Cytotoxicity Screening on a Test Cancer BioChip

The Test Cancer BioChip (Falcon Genomics, Inc.) was designed using the first-generation CBCS (CBC-1), as previously described [19]. It evaluated the cytostatic effects of the above-mentioned siRNAs in quintuplicate. On each CBC-1, we tested siRNAs targeting nine different genes (five replicates each) and included a no siRNA or non-targeting siRNA control (five replicates). Each well was 3 mm in diameter and 1 mm deep. Anchorage-independent growth was obtained by using soft agar as a base matrix (0.8%) to inhibit cellular attachment (Figure 1, step 1). In step 2 siRNA in 0.2% agar was added to each well followed by the cells mixed with 0.4% agar (step 3). After application of cells (500–700 cells/well), the CBC-1 was incubated at 37°C with 5% CO_2_ for up to 15 days until distinct colonies could be observed using an inverted microscope. The cells on the CBC-1 were starved overnight to allow for siRNA transfection and then covered with cell-specific medium one day following application of the cells. Each slide was fed with cell-specific media twice a week thereafter. Colony growth was quantified by imaging individual CBC-1 wells at a series of time points: 2, 7 or 8, and 14 or 15 days post seeding on the CBC-1 for MCF7 cells, and 2, 10, and 15 days post seeding for the SK-BR-3 cells. In order to capture the three-dimensional nature of the growth, a series of images was taken along the z-axis for each well at each time point using a 4 × microscope objective. After 14 or 15 days on the CBC-1, cells were stained for viability using MTT (Invitrogen), dissolved in PBS.

### Data Analysis

Macros written in Image J were employed for image analysis. A z-stack of minimum intensities was created for each well at each time point. A mask was then produced using an appropriate threshold and used to obtain cell count and cell size distribution. A number of parameters were assessed in order to completely evaluate the effects of siRNA on cell growth. These include total cell count and change in cell count, which were measured to identify siRNA that completely killed cells, and change in average cell size that was calculated to determine siRNA causing retardation in growth. Change in average cell size was calculated between day 2 and each of the other time points and expressed as percent of controls on each CBC-1. Measured particles were considered to be colonies if they were larger than average cell size at day 2 plus two standard deviations. Relative change in colony number was calculated by subtracting the number of colonies between day 2 and later time points, normalizing that number to total cell counts at day 2, and expressing it relative to controls. The main reason for measuring the change in cell size and numbers between day 2 and later time points was to eliminate the effect of cell clumps or overlapping cells in each well at day 2. For MCF7 and SK-BR-3 cells, the colony count by day 14 or 15 was determined to be inaccurate due to colonies becoming so large that they began to merge or overlap and was therefore not used for further analysis.

Doubling time was then calculated to determine siRNA that affected growth rate while cells were growing exponentially, and growth curves were drawn to assess effects on growth rate over time. We calculated doubling time, t_d_, using the equation,
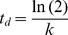
(1)where k is a growth rate constant. This rate constant was calculated by solving an exponential growth equation,
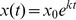
(2)where x is the total area covered by cells, x0 is the initial area covered by cells (day 2), and t is the elapsed time. This equation was solved using day 7 or 8 data for MCF7 and day 10 for SK-BR-3 cells since by day 14 or 15 colonies began to merge and overlap, and thus the area covered by cells was no longer increasing exponentially. Doubling time was normalized to controls on each individual slide.

In order to determine the screening capability of the CBCS, and thus validate the assay, a screening window coefficient, Z’, was calculated using the formula described by Zhang, et al (Equation 3) [20].
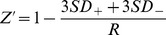
(3)SD_+_ is the standard deviation of the positive controls, SD_−_ is the standard deviation of the negative controls, and R is the dynamic range calculated as the absolute value of the difference between the means of the positive and negative controls. ESR1 siRNA was chosen as a positive control for the MCF7 screens, as it is well known that suppressing ESR1 expression in MCF7 cells inhibits growth and colony formation [21], [22], [23], and ERBB2 siRNA was applied as a positive control for SK-BR-3 cells as it is known to suppress their growth [21], [24]. Non-targeting siRNA and no siRNA were used as negative controls.

## Results

### Plating and Transfection Efficiency of MCF7 and SK-BR-3 Cells on the Test Cancer BioChip

We tested growth of two breast cancer cell lines: MCF7 and SK-BR-3. Both cell lines express most genes targeted on the Test Cancer BioChip at comparable levels, with the exception of ESR1, PGR, SCUBE2, CCNB1, and IGF1R being higher in MCF7 cells and GRB7 and ERBB2 being elevated in SK-BR-3 (data not shown). Thus, we determined whether silencing these genes would result in cell-line specific suppression of growth on the CBC-1.

We first assessed whether these cells would grow and form colonies on the CBC-1 (plating efficiency) and incorporate siRNA (transfection efficiency). We obtained plating efficiency of 39%±1% for MCF7 cells and 25%±1% for SK-BR-3 cells. Transfection efficiency, which was determined by measuring Accell Green fluorescence intensity levels, was found to be 79%±4% for MCF7 cells and 83%±3% for SK-BR-3 cells (Figure S1). These results show that we can grow both cell lines on the CBC-1 and a high percentage of the cells incorporate the underlying siRNA.

Transfection efficiency experiments also allowed us to determine whether there is cross-contamination between wells on the CBC-1. While we were able to observe strong fluorescence signal in the wells containing Accell Green siRNA, there was no fluorescence in the adjacent control wells. Figure S1 shows percentage of MCF7 and SK-BR-3 cells exhibiting fluorescence signal and representative images illustrating strong fluorescence signal in presence of Accell Green siRNA and the absence of signal in presence of control non-targeting siRNA. This verifies that the siRNAs were sufficiently immobilized by the agar and thus cross-contamination did not occur on the CBC-1.

### Evaluation of Control siRNA Effects on MCF7 Cells

**Figure 2:**
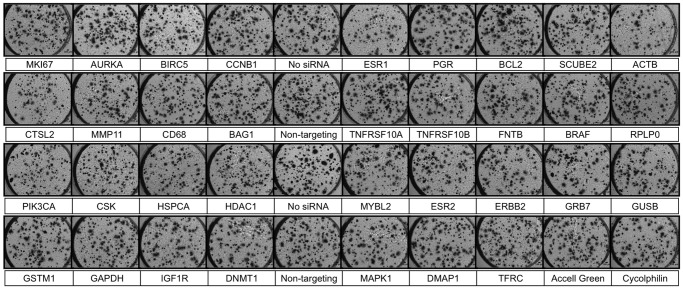
Representative images showing MCF7 colonies growing on the Test Cancer BioChip in presence of individual siRNAs. Cells were stained with MTT after 15 days on the CBC-1. Live colonies take up the dye and thus appear dark and slightly larger due to the formation of formazan crystals. Each well is 3 mm in diameter.

The Test Cancer BioChip was designed to simultaneously determine the effects of 40 different siRNAs in quintuplicate. We tested the growth of MCF7 cells under these conditions. Images were obtained at day 2 and once a week thereafter to monitor colony growth in 3D. At the end of this incubation period, live colonies were stained with 3-(4,5-Dimethylthiazol-2-yl)-2,5-diphenyltetrazolium bromide (MTT). Figure 2 shows representative merged z-stack images obtained at day 15 after culture of these cells on the CBC-1. Live colonies appear dark blue.

A number of parameters, including number of cells, average cell size, and the total area occupied by cells, were assessed to evaluate the effects of siRNA on cell growth on the CBC-1 at different time points. Percentage of cells forming colonies was also evaluated, growth curves were plotted, and doubling time was calculated. We employed the combination of these metrics to quantify effects of each siRNA on cell growth and thus identify potential gene targets for therapy.

**Figure 3:**
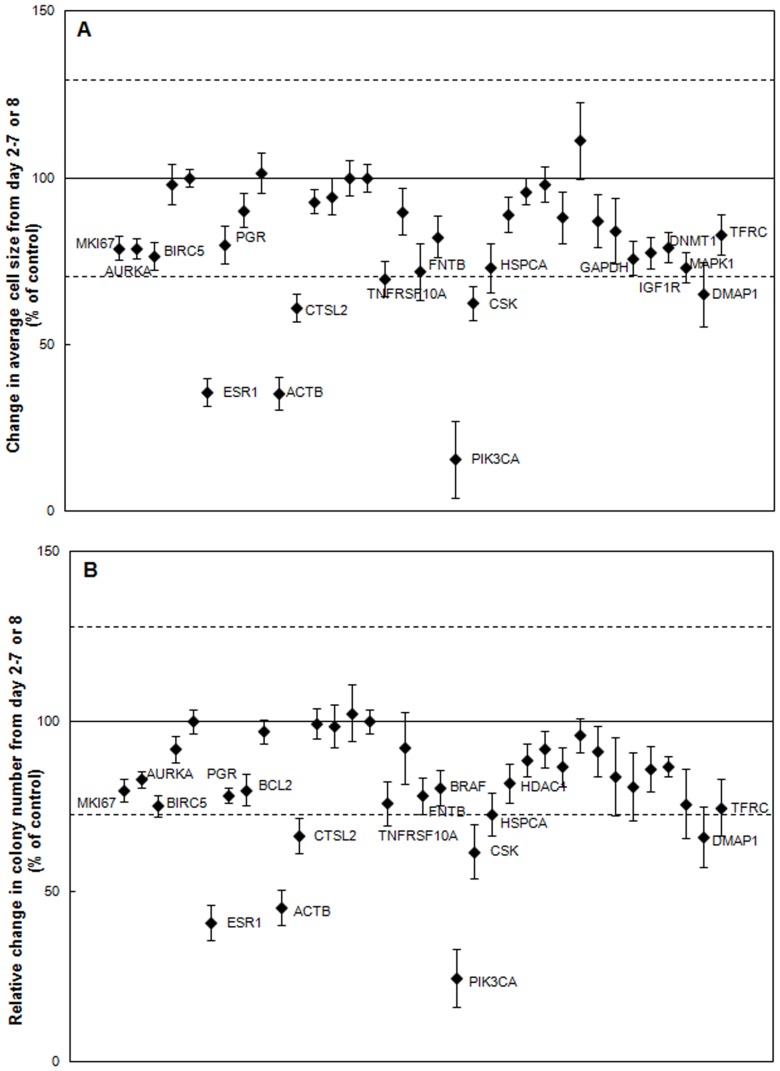
Identification of inhibitors of MCF7 cell growth on the CBC-1. A) Change in average MCF7 cell size from day 2–7 or 8 normalized to control (n = 6–11). B) Relative change in MCF7 colonies between day 2–7 or 8 normalized to control (n = 6–11). Labeled siRNAs are significantly different from control in A & B (p<0.05). Dashed lines represent two standard deviations away from the control mean.

For the Test Cancer BioChip, the negative controls employed included non-targeting, ACTB, GUSB, GAPDH, RPLP0, TFRC and cyclophilin siRNA as well as no siRNA. Using ANOVA followed by appropriate t-tests, we determined that ACTB had a significant effect on MCF7 cell growth (Figures 2 and 3) and thus could not be used as a control in this assay. Other siRNAs, including GAPDH and TFRC, produced minor effects. Thus, these siRNAs were not used as controls in this study and all data was normalized to no siRNA and non-targeting siRNA on each CBC-1.

We evaluated the reproducibility of the Test Cancer BioChip by performing two separate screens testing the effects of all targeted siRNAs on MCF7 growth. A Pearson correlation coefficient between the two MCF7 screens was calculated using each of the tested metrics including change in average cell size from day 2–7 or 8 (r = 0.8) and change in relative colony number from day 2–7 or 8 (r = 0.8). Overall, strong correlation was found between the screens.

We then evaluated the screening power of the CBC-1 using ESR1 siRNA as a positive control for the suppression of MCF7 cell growth. We found that ESR1 siRNA did significantly suppress MCF7 growth and colony formation on the CBC-1, reducing growth in average cell size to 36%±4% and 40%±4% of control from day 2–7 or 8 (Figure 3A) and day 2–14 or 15 (data not shown), respectively. Reduction in number of MCF7 colonies formed between day 2 and 7 or 8, to 41%±4% of control (Figure 3B), and increase in doubling time, to 265%±38% of control, were also observed with ESR1 siRNA (Table 1). The screening window coefficient, Z’-factor [20], was calculated for several screens using ESR1 siRNA as a positive control for MCF7 cells, and a Z’ equal to 0.3, indicating that a Z’-factor suitable for screening could be obtained.

**Table 1:** Doubling Time of MCF7 and SK-BR-3 cells in presence of different siRNAs on the CBC-1 (% of control).

	Average doubling time (% of control)	
siRNA	MCF7	SK-BR-3
ESR1	265±38*	142±35
ACTB	No fit	236±54*
CTSL2	141±12*	66±6**
FNTB	144±8*	94±11
PIK3CA	No fit	182±34*
CSK	164±29*	108±13
DMAP1	206±47*	70±6
SCUBE2	101±6	144±18*
ESR2	112±7	150±15*
ERBB2	121±20	No fit

* indicates siRNA causing a significant suppression (longer doubling time than control).

** indicates siRNA stimulating growth (significantly shorter doubling time than control). In some cases the data did not fit the exponential growth model, showing almost complete suppression. Only siRNAs that had the most significant effects in this study are shown.

### Identification of siRNA Capable of Inhibiting Anchorage-independent Growth of the Hormone Receptor Positive MCF7 Breast Cancer Cell Line on the Test Cancer BioChip

**Figure 4:**
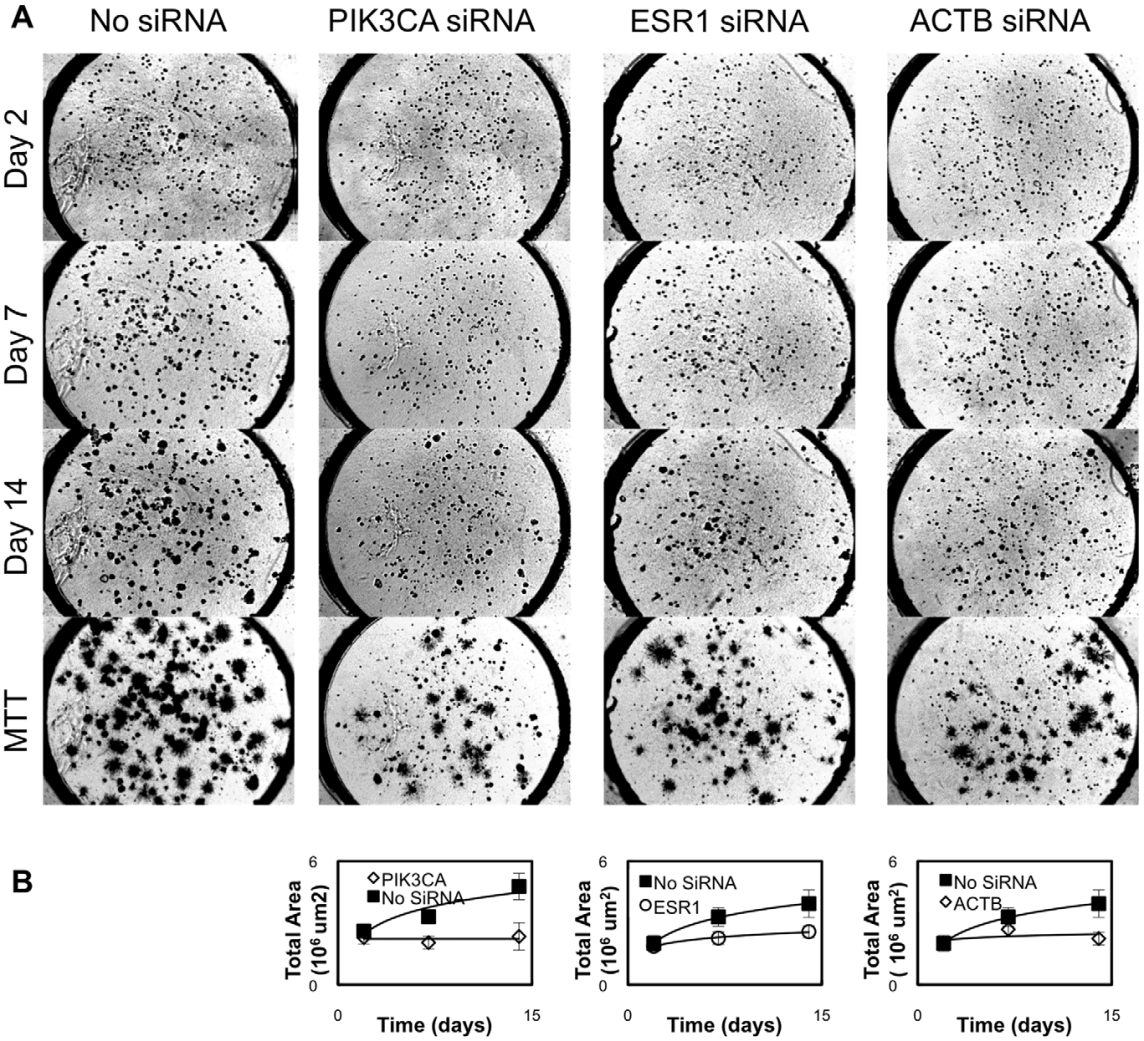
Time course of MCF7 colony suppression by PIK3CA, ESR1, and ACTB siRNA as compared to control no siRNA on the CBC-1. A) Representative images showing MCF7 colony formation over time in the presence of no siRNA, PIK3CA, ESR1, and ACTB siRNA. Live colonies are stained in the MTT images (stained on day 14) and appear dark and slightly larger due to the formation of formazan crystals. Each well is 3 mm in diameter. B) Growth curves show the change in total area covered by cells vs. time for these siRNAs and illustrate significant suppression caused by PIK3CA, ESR1, and ACTB siRNAs.

After validating the assay using appropriate controls, we examined the effect of the tested siRNAs on MCF7 colony formation (see Figure 2 for representative images). We determined the change in average cell size (Figure 3A) and relative change in colony number (Figure 3B) between day 2 and later time points for each well. Of the tested siRNAs, those targeting ESR1, PIK3CA, or ACTB caused the largest suppression in colony number and average size. Representative images and growth curves showing the suppression of growth over time caused by these siRNAs compared to that of controls are shown in Figure 4A & B. Colonies in the MTT stained images appear larger than their actual size due to the deposition of formazan crystals around the cells. Growth curves (Figure 4B) for MCF7 cells in presence of either ACTB or PIK3CA siRNA did not fit the exponential model. While we were able to observe that ESR1 siRNA increased doubling time to more than 250% of control, we could not measure doubling time for PIK3CA and ACTB siRNA (Table 1). These siRNAs almost completely suppressed growth to the point that the cells were no longer growing exponentially. These results show that ESR1, PIK3CA, and ACTB siRNA significantly suppress anchorage-independent growth of MCF7 cells.

Other siRNAs caused smaller but statistically significant suppression of relative colony number and average size, with increase in doubling time. Those include CTSL2, CSK, and DMAP1 siRNAs, which suppressed colony size and number by more than two standard deviations from the control mean. Other siRNAs caused even smaller, but statistically significant suppression in average size and relative colony number (Figure 3), with small but statistically significant increase in doubling time (Table 1). These results show that the Test Cancer BioChip can identify siRNAs that inhibit anchorage-independent growth of MCF7 cells to different degrees.

### Identification of siRNA Capable of Inhibiting Anchorage-independent Growth of the HER2 Positive SK-BR-3 Breast Cancer Cell Line on the Test Cancer Biochip

**Figure 5:**
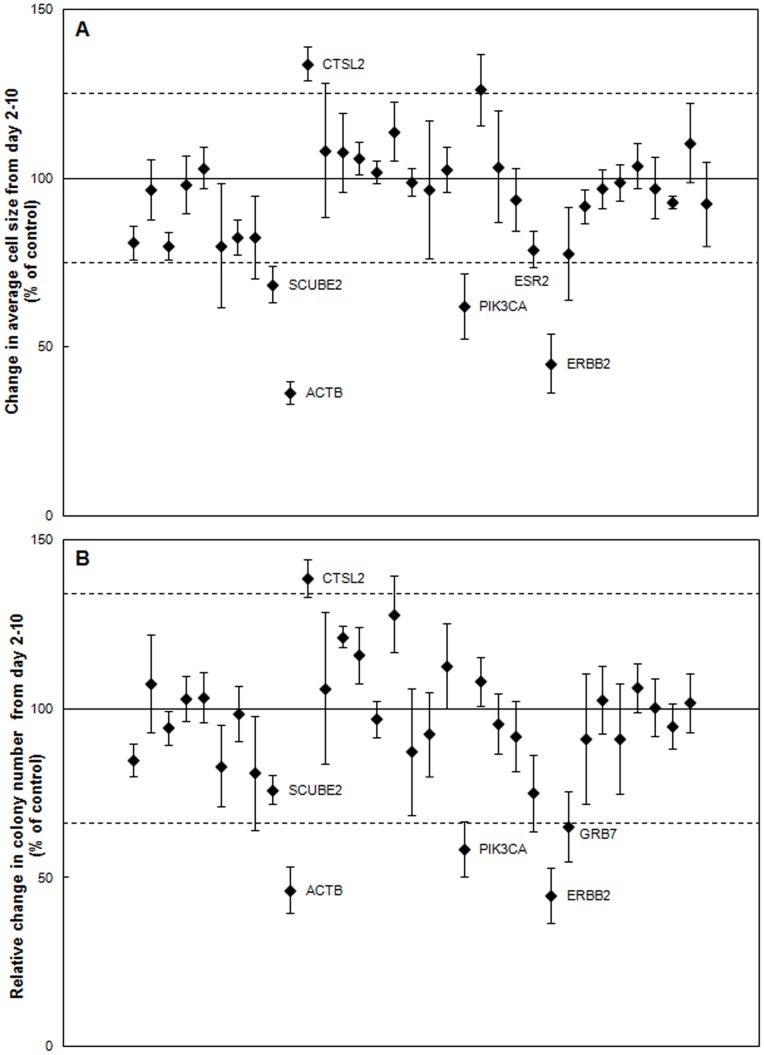
Identification of inhibitors of SK-BR-3 cell growth on the CBC-1. A) Change in average SK-BR-3 cell size from day 2–10 normalized to control (n = 3–5). B) Relative change in SK-BR-3 colonies between day 2–10 normalized to control (n = 3–5). Labeled siRNAs are significantly different from control in A & B (p<0.05). Dashed lines represent two standard deviations away from the control mean.

We then determined the efficacy of these siRNAs to inhibit SK-BR-3 colony formation and growth on the Test Cancer BioChip and assessed cell type-specific responses. The positive control for these cells, ERBB2 siRNA, caused a decrease in SK-BR-3 colony number and size. It reduced average cell size change from day 2 to 10 to 45%±9% of control (Figure 5A) and relative colony number change from day 2 to 10 to 45%±8% of control (Figure 5B). While SK-BR-3 cells transfected with either no siRNA or non-targeting control grew exponentially on the CBC-1, SK-BR-3 cells transfected with ERBB2 siRNA did not. Therefore, doubling time could not be calculated for these cells (Table 1). We used the average cell size change to calculate the screening window coefficient (Z’-factor) for this assay. We found that the screening window using ERBB2 siRNA as positive control was 0.

Comparison of the results from the Test Cancer BioChip using MCF7 and SK-BR-3 cells showed cell-specific effects of several siRNAs. While ERBB2 siRNA suppressed SK-BR-3 growth, it had no effect on MCF7 cells (Figure 3, Table 1). In addition, growth of SK-BR-3 cells was not affected by ESR1 siRNA (Figure 5, Table 1), showing a cell type-specific response.

Immediate effects of siRNA on SK-BR-3 growth on the Test Cancer Biochip were observed at day 2 for ERBB2, ESR2, CSK, CTSL2, and BRAF siRNAs. While ERBB2 and ESR2 siRNAs suppressed cell counts at day 2, CTSL2 siRNAs caused an increase (data not shown). These effects were maintained at later time points. At day 2, BRAF siRNA also caused an initial suppression and CSK siRNA caused an initial increase. These effects, however, were not maintained.

**Figure 6:**
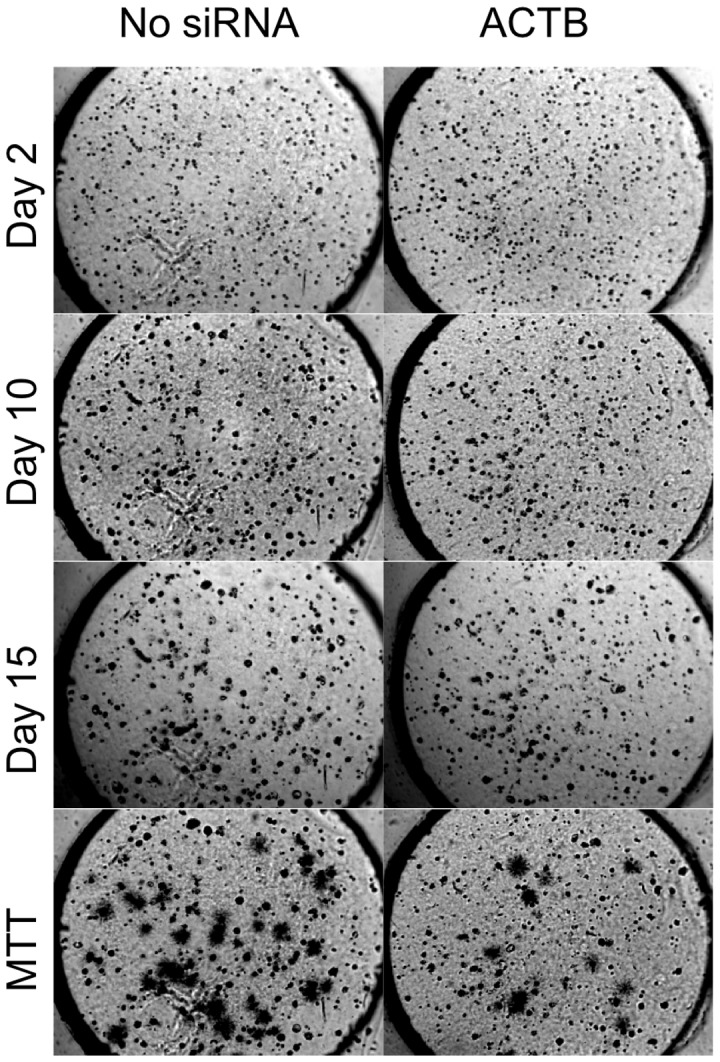
Representative images showing the suppression of SK-BR-3 cell growth over time caused by ACTB siRNA. Live colonies were stained with MTT on day 15. Each well is 3 mm in diameter.

At day 10, the strongest suppression of SK-BR-3 growth on the Test Cancer BioChip was observed using siRNAs for ACTB, PIK3CA, and ERBB2 (Figures 5A & B). Figure 6 shows representative images illustrating the suppression of colony formation over time caused by ACTB siRNA. The reduction in colony growth by this siRNA at day 10 was maintained at later time points. Other siRNAs, such as those targeting SCUBE2, ESR2, and GRB7 caused smaller but significant suppression in colony size and/or colony numbers, while CTSL2 siRNA caused a small but statistically significant increase in colony growth (Figures 5A & B). The siRNAs that inhibited colony formation also significantly increased SK-BR-3 doubling time (Table 1). The increased growth caused by CTSL2 siRNA was also evident from its significantly faster doubling time than control. While suppression of ACTB and PIK3CA inhibited growth of both SK-BR-3 and MCF7 cells, CTSL2 effects were opposite in the two cell lines.

In summary, we have employed the Test Cancer Biochip as a fast, one-step tool for the identification of inhibitors of anchorage-independent breast cancer cell growth in real time. Results from these studies showed cell type-specific effects of some siRNAs as well as effects that are unique to the 3D anchorage-independent nature of this assay.

## Discussion

In this paper, we have used a Test Cancer BioChip that contains siRNA for breast cancer targets and controls and determined their effects on anchorage-independent growth of hormone receptor positive (MCF7) and HER2 positive (SK-BR-3) breast cancer cell lines. When cultured on the CBC-1, both cell lines formed colonies, and a high percentage of cells incorporated the tested siRNA. Comparison of the percent of cells that formed colonies in the presence of all tested siRNAs revealed cell type-specific responses with some that are unique due to the 3D nature of the assay.

### Validation of Positive Controls on the Test Cancer BioChip

In our study, we identified PIK3CA siRNA to be a significant suppressor of anchorage-independent growth of both MCF7 and SK-BR-3 cells on the CBC-1. These results correlate well with findings from previous siRNA screens performed on these cells while they were growing on flat surfaces. For example, a recent study by Brough and coworkers [21], which screened the effects of various siRNAs on a number of breast cancer cell lines, also found PIK3CA siRNA to significantly decrease the viability of SK-BR-3 and MCF7 cells. In addition, the importance of PIK3CA for the survival of MCF7 cells was shown in a siRNA screen of the effects of a library of kinases on the growth of attached MCF7 cells on a flat surface [25]. The agreement between our data and previously published data on the effects of PIK3CA on breast cancer cell lines helps to validate the efficacy of the CBC-1 for screening of targeted therapies for breast cancer.

Along with inhibitors common to both tested cell lines, we found the Test Cancer BioChip to be capable of identifying cell-specific effects of certain siRNAs. For example, we observed significant inhibition of SK-BR-3 growth caused by ERBB2 siRNA but saw no effect of this siRNA on MCF7 cells. This is in agreement with other studies that have shown ERBB2 to be essential for SK-BR-3 growth and survival including high throughput siRNA screens [21], [24]. The observed lack of effect of ERBB2 siRNA on MCF7 cells also agrees with previous studies [25]. Inhibiting GRB7, another gene that has been shown to be essential for the growth of ERBB2-positive cell lines including SK-BR-3 [26], also caused significant suppression in colony number for these cells. MCF7 growth on the CBC-1, while not affected by inhibition of ERBB2, was significantly suppressed by ESR1 siRNA. This observation also agrees well with past studies, including high throughput siRNA screens, which showed the inhibitory effects of silencing ESR1 on MCF7 growth on flat surfaces [21], [22], [23]. These findings confirm the ability of the Test Cancer Biochip to detect cell-specific inhibitors of anchorage-independent growth. This also suggests that positive controls for CBCS screens have to be selected in a patient-specific manner.

### Identification of Genes that Exhibit Anchorage-independent Specific Effect on Breast Cancer Cell Growth

One of the interesting effects we found on the Test Cancer BioChip was the suppression of anchorage-independent growth of both tested cell lines by ACTB siRNA. In many assays, ACTB is used as a negative control since it is a major component of the cytoskeleton. Its suppression in most siRNA screens for cells growing on flat surfaces did not alter cellular phenotypes. However, ACTB has been reported to play a role in the migration, motility, and invasion of mammary epithelial cells including breast cancer cells [27]. SiRNA targeting ACTB has been shown to reduce migration of MCF10A breast epithelial cells in a high throughput siRNA screen [28], and down-regulation of AIB1, an adaptor protein involved in actin reorganization and polymerization, suppressed migration, invasion, proliferation and colony formation in MDA-MB-231 breast cancer cells [29]. Many chemotherapeutic agents currently being used in the clinic or being investigated rely on their ability to disrupt the cytoskeleton. Cisplatin, a drug that is used in treating various cancers including breast cancer, disrupts the formation of microtubules, causes collapse and aggregation of tubule and filament networks in the cytoskeleton, and induces apoptosis [30]. Vinorelbine and Vinfluine, other chemotherapy agents used in treating breast cancer, also work by disrupting microtubule formation in the cytoskeleton [31]. A recent study investigated the effects of latrunculin B and pectenotoxin-2, cytotoxic agents derived from natural origins that inhibit actin polymerization, and jasplakinolide, which prevents actin depolymerization, on MCF7 cells. This study found that disrupting actin lead to G2 arrest and thus apoptosis in MCF7 cells [32]. Another recent study found that, when treated with agents that disturbed actin, the cytoskeleton of MDA-MB-231 breast tumor cells was altered, thus disrupting cell-generated force [33]. While ACTB siRNA is commonly used as housekeeping control in siRNA screens and was found to have no effects on cell growth in other screens performed with attached cells on flat surfaces [1], [34], our results suggest that it has a significant inhibitory effect on the 3D anchorage-independent growth of breast cancer cells. The current study is the first report of ACTB siRNA suppressing anchorage-independent growth. While many chemotherapeutic agents currently act by disrupting the cytoskeleton, directly targeting ACTB with siRNA may have similar cytotoxic effects on the tumor cells. Although ACTB siRNA inhibited colony growth in both cell lines tested in this study, we found that it affected some, but not all, breast cancer patient cells when tested on the CBCS (manuscript in preparation). These findings stress the utility of the 3D anchorage-independent platform provided by the CBCS in identifying targets for breast cancer therapy as well as the necessity of choosing patient specific therapies.

Another unique finding from these screens was that inhibiting CTSL2, one of the tested invasion genes, suppressed MCF7 growth on the Test Cancer Biochip but stimulated growth of SK-BR-3 cells. Cathepsins are lysosomal cysteine proteases that are involved in extracellular matrix degradation [35]. Their intracellular activity also is thought to play a role in cancer progression [36]. Cell type-specific responses to silencing this gene have been observed. Two RNAi screens found that CTSL2 siRNA did not have any effect on MCF10A breast epithelial cells or human embryonic stem cells [28], [34]. Other studies have found that inhibition of cathepsin L significantly reduced tumor invasion and proliferation, increased cell death [37], [38], and prevented resistance to chemotherapy in animal models [39], [40]. While most previous studies were performed either on attached cells on flat surfaces or in mouse models, inhibition of cysteine proteases, including cathepsin L, in soft agar assays significantly reduced colony formation and growth of ras-transformed NIH3T3 cells [38] and MCF7 cells [41]. The observed stimulation of SK-BR-3 growth on the CBC-1 with CTSL2 siRNA was surprising considering that inhibition of cathepsin L has been shown to reduce SK-BR-3 proliferation on flat surfaces [42]. It is likely that this effect is mediated by the function of cathepsins in the cytosol, where they appear to play a role in initiating apoptosis [43]. This may explain why inhibition of CTSL in tumor mouse models was associated with increased intestinal and epidermal tumor progression, but decrease of pancreatic tumors [36], [39], [44]. This suggests that the cell-specific effects of cathepsin L inhibition could only be observed in a setting that has high in vivo translatability. The 3D, anchorage-independent nature of the CBCS, however, allowed us to detect this phenomenon in our screen using MCF7 and SK-BR-3 cells. The observation of such a cell type-specific response to CTSL2 siRNA stresses the need for screening of individual patient cells to identify target therapies, one of the unique capabilities of the CBCS. In addition, the observation of effects on the Test Cancer BioChip, but not in other cell based assays, demonstrates the advantage of the more clinically translatable anchorage-independent growth platform provided by the CBCS.

Two other siRNAs caused large suppressions in MCF7 colony formation and growth (reduced colony size and relative colony number by more than two standard deviations of the mean). These include CSK and DMAP1 siRNAs. CSK siRNA, which targets c-Src, suppressed MCF7 growth, but not SK-BR-3 growth, on the CBC-1. A previous study showed that silencing c-Src expression in MCF7 cells significantly reduced cell migration and proliferation [45]. High throughput RNAi screens, however, did not find any effect of inhibition of CSK on HCC1954, MCF10A or HMEC cells [1], [28]. This finding supports the likelihood of a cell type-specific response to this siRNA and stresses the need for testing of individual patient cells. Targeting DMAP1 also caused significant inhibition of MCF7 growth on the CBC-1. DMAP1 is known to form a complex with DNMT1 and repress transcription in tumor cells [46]. DNMT1 siRNA caused a smaller but statistically significant suppression of MCF7 growth in our assay. Previous studies have shown that inhibiting DNMT1 hindered growth of MCF7 cells [47] and HMECS [1]. MCF7 suppression by DNMT siRNA was previously seen both in growth of attached cells and in an anchorage-independent fashion [47].

Although we focused on the siRNAs that caused the most suppression, as those genes would be the most likely candidates for therapeutic targets, we were also able to identify more minor suppressors using the Test Cancer BioChip. Inhibition of genes including AURKA [21], MKI67 [2], BIRC5 [48], FNTB [49], BRAF [50], and IGF1R [51], [52] has previously been shown to suppress MCF7 cell growth. We observed only mild suppression of anchorage-independent growth of MCF7 cells caused by these siRNAs. These results suggest that the effects of inhibiting certain genes may be dependent on either growth on flat surfaces or cell-cell contact. Thus, these observations further emphasize the relevance of screening effects of compounds on anchorage-independent cell growth.

It is highly unlikely that the observed responses to siRNA on the CBC-1 are caused by off-target effects. Such unspecific effects of siRNA have been previously attributed to a variety of factors, including the transfection agent used for siRNA introduction into the cell [53], interferon pathway activation caused by large siRNA sequences [54], [55], and RISC-dependent off-target effects. We have eliminated these problems by:

1. Using Accell siRNA pooled sequences from ThermoFischer Dharmacon. These siRNA sequences were designed to significantly reduce off-target effects by employing:
  - Bioinformatics: According to Dharmacon, seed region analysis was performed on Accell siRNA sequences to eliminate motifs that could cause 3′UTR miRNA-like interactions and toxicity [56]. All siRNA sequences were designed to increase specificity to the target gene with no interferon pathway activation.
  - Chemical modifications: Chemical modifications on Accell siRNA eliminate the need for transfection reagents, which are the most common cause for off-target effects [57]. In addition, specific nucleotides on the antisense strand were identified to be responsible for off-target effects and modified for improved specificity.
  - siRNA pooling: Off-target effects of siRNA are concentration dependent, and pooling of 4 different siRNA sequences in our studies dilutes the concentration of toxic sequences.
2. Testing up to 40 different Accell siRNA pools on the CBC-1, 5 of which target control housekeeping genes. Most sequences did not alter colony growth as compared to control.
3. Screening the effect of these siRNAs on two different cell lines. We have observed cell-specific effects on colony growth by positive control siRNAs (ESR1 and ERBB2). These cell-type specific responses could not be due to off-target effects.

This study demonstrates that the CBCS is a powerful tool for determining cell type-specific responses to silencing of individual genes. We identified ACTB siRNA to be a novel suppressor of MCF7 and SK-BR-3 colony formation in a 3D anchorage-independent manner, while previous studies did not find effects of this siRNA on cells growing on flat surfaces. We also found a cell type-specific response to CTSL2 siRNA. These observations stress the utility of the more clinically relevant 3D platform provided by the CBC-1 for identifying targets for breast cancer therapy as well as the patient specificity in choosing an effective therapy.

The 3D, anchorage-independent platform provided by the CBCS allows for the identification of effects such as these that have not been observed in assays conducted with attached cells on flat surfaces. Thus, the inhibitors identified in this assay may have improved clinical translatability. Another advantage of the CBCS is that it allows for targeting of a large number of genes using a one-step assay. Moreover, the ability of the CBCS to quantify real time colony formation and growth allows us to identify siRNAs that cause immediate effects as well as those which cause downstream effects. Thus, the CBCS offers many unique features, which facilitate the identification of gene targets for novel, individualized cancer therapy. .

## Supporting Information

Figure S1 **Verification of the absence of cross-contamination on the CBC-1.** A) Percentage of cells exhibiting fluorescence signal (mean ± SEM) for control MCF7 cells and those transfected with Accell Green siRNA, as well as representative images, show absence of fluorescence in control wells adjacent to those containing Accell Green. B) Percentage of cells exhibiting fluorescence signal (mean ± SEM) for control SK-BR-3 cells and those transfected with Accell Green siRNA, as well as representative images, show absence of fluorescence signal in control wells adjacent to those containing Accell Green.
(PDF)

Table S1 **siRNA sequences and accession numbers used in this study.**
(XLSX)
