# Supplementary figures and images for: Validation of the Cancer BioChip System as a 3D siRNA Screening Tool for Breast Cancer Targets
Source: PLoS One. 2012 Sep 26;7(9):e46086. doi: 10.1371/journal.pone.0046086 (PMC3458802; doi:10.1371/journal.pone.0046086)

Figure S1

A

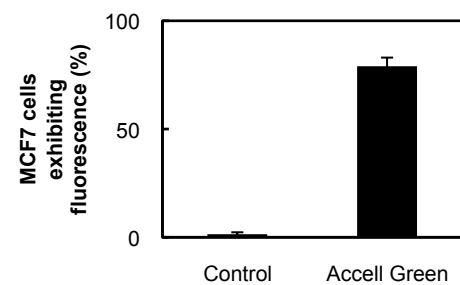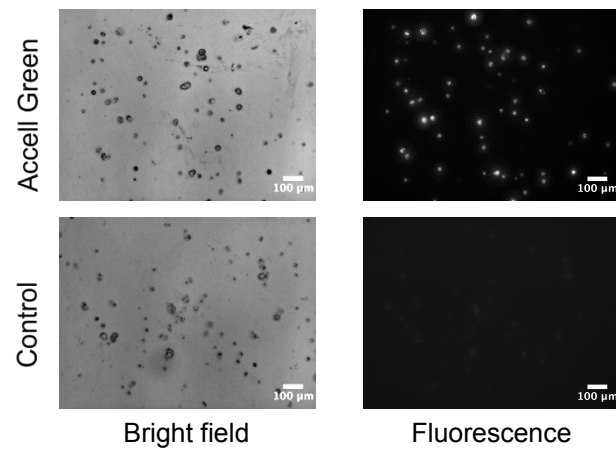

B

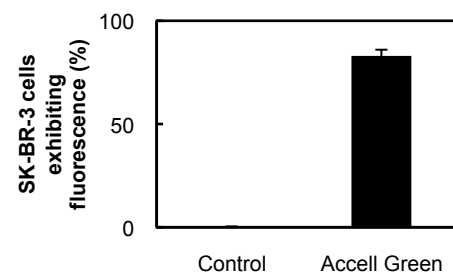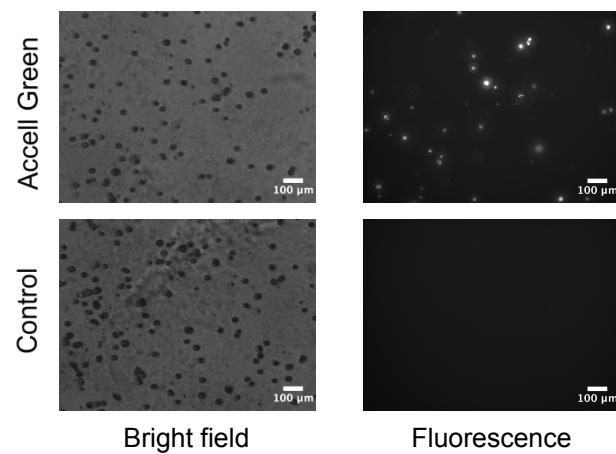

Supplement: Figure S1 — Verification of the absence of cross-contamination on the CBC-1. A) Percentage of cells exhibiting fluorescence signal (mean ± SEM) for control MCF7 cells and those transfected with Accell Green siRNA, as well as representative images, show absence of fluorescence in control wells adjacent to those containing Accell Green. B) Percentage of cells exhibiting fluorescence signal (mean ± SEM) for control SK-BR-3 cells and those transfected with Accell Green siRNA, as well as representative images, show absence of fluorescence signal in control wells adjacent to those containing Accell Green. (PDF) [file pone.0046086.s001.pdf]
